# Purinergic control of inflammation and thrombosis: Role of P2X1 receptors

**DOI:** 10.1016/j.csbj.2014.11.008

**Published:** 2014-11-28

**Authors:** Cécile Oury, Christelle Lecut, Alexandre Hego, Odile Wéra, Céline Delierneux

**Affiliations:** Laboratory of Thrombosis and Hemostasis, GIGA —Cardiovascular Sciences, University of Liège, Liège, Belgium

**Keywords:** Thrombosis, Inflammation, P2X receptor

## Abstract

Inflammation shifts the hemostatic mechanisms in favor of thrombosis. Upon tissue damage or infection, a sudden increase of extracellular ATP occurs, that might contribute to the crosstalk between inflammation and thrombosis. On platelets, P2X1 receptors act to amplify platelet activation and aggregation induced by other platelet agonists. These receptors critically contribute to thrombus stability in small arteries. Besides platelets, studies by our group indicate that these receptors are expressed by neutrophils. They promote neutrophil chemotaxis, both *in vitro* and *in vivo*. In a laser-induced injury mouse model of thrombosis, it appears that neutrophils are required to initiate thrombus formation and coagulation activation on inflamed arteriolar endothelia. In this model, by using P2X1−/ − mice, we recently showed that P2X1 receptors, expressed on platelets and neutrophils, play a key role in thrombus growth and fibrin generation. Intriguingly, in a model of endotoxemia, P2X1−/ − mice exhibited aggravated oxidative tissue damage, along with exacerbated thrombocytopenia and increased activation of coagulation, which translated into higher susceptibility to septic shock. Thus, besides its ability to recruit neutrophils and platelets on inflamed endothelia, the P2X1 receptor also contributes to limit the activation of circulating neutrophils under systemic inflammatory conditions. Taken together, these data suggest that P2X1 receptors are involved in the interplay between platelets, neutrophils and thrombosis. We propose that activation of these receptors by ATP on neutrophils and platelets represents a new mechanism that regulates thrombo-inflammation.

## 1 Introduction: hemostasis and thrombosis

Hemostasis is the process that maintains the integrity of a closed circulatory system after vascular damage. Vessel wall injury and the extravasation of blood from the circulation rapidly initiate events in the vessel wall and in the blood that seal the breach. Circulating platelets are recruited to the site of injury, where they become a major component of the developing thrombus; blood coagulation is initiated by endothelium-expressed tissue factor and leads to the generation of thrombin and fibrin. Under normal conditions, regulatory mechanisms restrain thrombus formation both temporally and spatially [1]. When pathologic processes overwhelm the regulatory mechanisms of hemostasis, excessive quantities of thrombin form, initiating thrombosis. Thrombosis is a critical event in the arterial disease progression and is associated with myocardial infarction and stroke, accounting for considerable morbidity and mortality [2].

## 2 Platelet P2 receptors

Adenosine diphosphate (ADP) plays crucial roles in the physiological process of hemostasis and in the development and extension of arterial thrombosis. By itself ADP is a weak agonist of platelet aggregation inducing only reversible responses as compared to strong agonists such as thrombin or collagen. However, due to its presence in large amounts in the platelet dense granules and its release upon activation at sites of vascular injury, ADP is an important so-called secondary agonist amplifying most of the platelet responses, which contributes to the stabilization of the thrombus [3–5]. More recent studies indicate that ATP, co-released with ADP, should be considered alongside ADP and thromboxane A_2_ as a significant secondary platelet agonist [6,7].

The receptors for extracellular nucleotides belong to the P2 family which consists of two classes of membrane receptors: P2X ligand-gated cation channels (P2X1–7) and G protein-coupled P2Y receptors (P2Y1,2,4,6,11,12,13,14) [8]. Starting from the concept of a unique P2T receptor (T for thrombocyte) originally postulated on the basis of pharmacological data, a model of three platelet P2 receptors progressively emerged. These are the P2X1 cation channel activated by ATP and two G protein-coupled receptors, P2Y1 and P2Y12, both activated by ADP [4,9]. Each of these receptors has a specific function during platelet activation and aggregation, which logically has implications for their involvement in thrombosis.

Large-scale clinical trials have demonstrated the beneficial effects of thienopyridines, targeting P2Y12 receptors, in the prevention of major cardiac events after coronary artery stenting and in the secondary prevention of major vascular events in patients with a history of cerebrovascular, coronary or peripheral artery disease. More recently, new classes of P2Y12 inhibitors have been developed in order to circumvent clopidogrel limitations (*i.e.* variability of platelet inhibitory effect) for the management of ischemic coronary syndromes [10–12].

## 3 Platelet P2X1 receptors

The P2X1 receptor belongs to a family of ATP-gated ion channels, comprising seven mammalian receptor subunits (P2X1–7) that assemble to form a variety of homotrimeric and heterotrimeric receptors widely expressed in the body. Each P2X subunit contains two transmembrane domains, intracellular amino and carboxy termini and a large extracellular ligand-binding loop. P2X receptors vary in their kinetics of desensitization and pharmacology, although all are activated by the physiological ligand ATP [13]. The function of P2X1 receptors in neurogenic smooth muscle contraction, and in thrombosis has been well documented [14–17]. Mutagenesis studies identified residues important in agonist action, the inter-subunit nature of the binding site, the location of the channel gate, and interactions between the transmembrane regions [18–21]. The crystallization of a zebrafish P2X4 receptor in both resting and ATP-bound open states [22,23] demonstrated extensive conformational changes in the receptor associated with agonist binding and channel gating. Individual P2X receptor subunits have been described by analogy to a dolphin, with the ATP binding site formed predominantly from residues in the upper and lower body regions of adjacent subunits. Agonist binding induces movement of the dorsal fin, left flipper, and the cysteine-rich head regions closing the ATP binding pocket. This movement is translated through the body region to the transmembrane regions and results in opening of the channel gate.

The P2X1 receptor plays an important role in thrombus formation especially under high-shear conditions. P2X1-deficient mice have no prolongation of bleeding time as compared to the wild-type mice, indicating that they conserve normal hemostasis [24]. In contrast, they display resistance to the systemic thromboembolism induced by the injection of a mixture of collagen and adrenaline and to localized laser-induced injury of the vessel wall of mesenteric arteries. Conversely, increased arterial thrombosis has been reported in the microcirculation of mice overexpressing the human P2X1 receptor [25]. The P2X1 antagonist NF449 [4,4′,4″,4‴-(carbonylbis(imino-5,1,3-benzenetriylbis-(carbonylimino)))tetrakis-benzene-1,3-disulfonic acid octasodium salt] has an inhibitory effect on platelet activation *ex vivo* and on thrombosis *in vivo* [26,27]. Platelet P2X1 receptor function can also be inhibited by using heat shock protein 90 inhibitors, which may be as effective as selective antagonists in regulating thrombosis [28].

About 10% of current flow through the P2X1 receptor is mediated by Ca^2 +^ [29]. These ion channels can therefore provide a significant source of direct Ca^2 +^ influx into the cell following activation, as well as causing membrane depolarization. The time course of ATP-evoked P2X1 receptor-mediated currents is concentration-dependent with low concentrations taking several seconds to reach a peak response, which can be sustained for > 30 s. In contrast, at maximal agonist concentrations, P2X1 receptor currents peak within tens of milliseconds and desensitize completely within seconds [30]. In platelets, P2X1-mediated increase in intracellular Ca^2 +^ leads to the activation of ERK1/2 MAPK and MLCK that phosphorylates myosin light chain (MLC), a process accompanying platelet shape change and degranulation [31]. P2X1 receptor signaling represents a significant pathway for early Ca^2 +^-mobilization following activation of a variety of major platelet receptors through both G-proteins and tyrosine kinases [6,32]. Furthermore, P2X1 receptors seem to play a pivotal role in the activation of aspirin-treated platelets by thrombin and epinephrine [33]. Since aspirin is used extensively to manage cardiovascular diseases and since, in clinical research, much attention has been focused on “aspirin resistance” (meaning treatment failure), the finding that P2X1 receptors can circumvent the action of aspirin on platelet stimulation by thrombin is of major importance. P2X1-mediated Ca2 + mobilization has been involved in platelet responses to microbial pathogen-associated molecular patterns acting through Toll-like receptor 2 [34], suggesting a role for P2X1 in platelet-dependent sensing of bacterial components. Moreover, such P2X1 signals would be resistant to endogenous platelet inhibiting agents, such as prostacyclin, which may be particularly important during early thrombotic or immune-dependent platelet activation [35].

These results clearly indicate that the P2X1 receptor might be considered as a potential target for antiplatelet strategies, with the interesting feature that P2X1 antagonists should be effective only at sites of severe stenosis where shear forces are very high, without having a deleterious effect on normal hemostasis.

## 4 Neutrophil P2X1 receptors

We recently showed that P2X1 receptors are also expressed on neutrophils [36]. P2X1 activation causes ROCK-dependent MLC phosphorylation, promoting cytoskeletal reorganization and neutrophil deformation during chemotaxis. Intriguingly, we found that P2X1 deficiency increases neutrophil NADPH oxidase activity [37]. Indeed, *ex vivo* stimulation of P2X1−/ − neutrophils with various stimuli, including bacterial formylated peptides, phorbol esters, and opsonized zymosan particles resulted in increased production of reactive oxygen species as compared to neutrophils isolated from wild-type mice. These results indicated that P2X1 would act to limit systemic neutrophil activation through a negative feedback loop, allowing them to migrate to the site of inflammation. In agreement with this proposition, intraperitoneal injection of a sub-lethal dose of lipopolysaccharide (LPS) in P2X1−/ − mice, led to increased release of plasma myeloperoxidase (MPO) concentration, an indicator of neutrophil systemic activation, as compared to wild type mice. In addition, peripheral P2X1−/ − neutrophils expressed higher levels of CD11b in response to LPS injection, reflecting their higher activation state. Concomitantly, we observed that the LPS-induced drop in platelet and lymphocyte counts were both worsened in the P2X1−/ − mice as compared to their wild type littermates. Immunohistochemistry and MPO activity assay revealed exaggerated neutrophil relocalization into the lungs of P2X1−/ − mice, where these cells formed large aggregates in the capillary lumen. Finally, intraperitoneal injection of a lethal dose of LPS, the P2X1−/ − mice exhibited shorter survival time than wild type mice, most likely as a consequence of enhanced neutrophil-dependent ischemic events and subsequent multiple organ failure. Notably, this phenotype was not associated with altered plasma levels of the main LPS-induced cytokines, TNF-α, IL-6, IL-1β, and INF-γ. Taken together, these findings support an important role for P2X1 receptors in the homeostatic regulation of circulating neutrophils and in their recruitment at the sites of inflammation/infection.

## 5 Platelet and neutrophil P2X1 receptors in thrombosis

Several studies indicate that besides their ability to kill pathogens, neutrophil activation promotes coagulation in the microcirculation, trapping invading pathogens in fibrin mesh, thereby restricting microbial dissemination [38]. Furthermore, in the absence of any bacterial challenge, the neutrophil serine proteases elastase and cathepsin G, together with externalized nucleosomes contribute to large vessel thrombosis. Nucleosomes form a platform on which neutrophil serine proteases coassemble with the anticoagulant tissue factor pathway inhibitor (TFPI), supporting TFPI degradation and unleashing suppression of factor Xa, thereby fostering fibrin generation. In line with a contribution of activated neutrophils to coagulation, we observed increased thrombin generation and shortened coagulation time in the plasma of LPS-treated P2X1−/ − mice as compared to wild-type littermates. In a model of laser-induced injury of cremaster muscle arterioles, Darbousset et al. recently showed that neutrophils accumulate at the site of injury before platelets, contributing to the initiation of thrombosis. Neutrophils recruited to the injured vessel wall express tissue factor (TF), thereby promoting coagulation and thrombus growth. In collaboration with Dubois' team, we recently found that P2X1 deficiency or antagonism impairs neutrophil recruitment and activation on inflamed arteriolar endothelia, platelet accumulation and fibrin generation [39]. Infusion of wild-type neutrophils in P2X1−/ − mice was sufficient to fully restore fibrin generation, whereas infusion of both wild-type platelets and neutrophils were required to allow normal thrombus growth. Thus, P2X1 expressed on neutrophils and platelets is required for thrombosis.

The data reported so far assumed that the effects of platelet and neutrophil P2X1 receptors are mediated by homotrimeric P2X1 receptors. It must be known that P2X1 can also interact with other P2X subunits, *e.g.* P2X5, to form heteromeric ion channels with distinct properties [40]. Though several studies indicate that only homomeric P2X1 receptors form ATP-gated ion channels in platelets [41–43], this may not be the case for neutrophils. Indeed, neutrophils express other P2X subtype mRNAs: P2X4, P2X5 and P2X7 [44–47]. However, the expression of functional P2X4 or P2X5 subunit containing receptors has never been confirmed and it appeared that human neutrophils do not express functional P2X7 receptors. To determine whether the effects reported in P2X1-deficient neutrophils could be due to changes in the stoichiometry of putative heterotrimeric P2X receptors requires further investigations.

## 6 Summary and outlook: P2X1 receptors in thrombo-inflammatory disorders

**Fig. 1:**
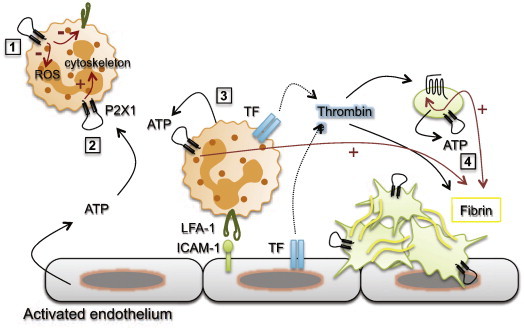
A role for platelet and neutrophil P2X1 receptors in thrombosis. Experimental data in mice indicate that activation of P2X1 receptors by extracellular ATP acts to maintain circulating neutrophil in a quiescent state (1), recruit neutrophil at the site of endothelial injury (2), and activate adhered neutrophils (3) and platelets (4), thereby promoting thrombus growth and fibrin generation. TF: tissue factor, ROS: reactive oxygen species.

In summary, our latest findings indicate that P2X1 receptors contribute to ATP-dependent thrombosis in mouse microcirculation by promoting early neutrophil and platelet recruitment and subsequent fibrin generation, locally, at sites of endothelial injury (Fig. 1). Upon systemic inflammatory challenge, P2X1 receptors would act to dampen the activation of circulating neutrophils, thereby limiting oxidative tissue damage and disseminated intravascular coagulation.

Targeting P2X1 receptors will not only inhibit platelets but also alter neutrophil function, and may therefore represent an innovative therapeutic strategy to prevent local thrombo-inflammation, only if neutrophil regulatory homeostasis is preserved. Future research should focus on the role of P2X1 receptors in the pathophysiology of thrombo-inflammatory disorders such as ischemic stroke. In stroke, thromboembolic occlusion of major or multiple smaller intracerebral arteries leads to focal impairment of the downstream blood flow, and to secondary thrombus formation within the cerebral microvasculature [48]. Pathologic platelet activity has been linked to cerebral ischemic events [49]. Therapeutic thrombolysis (t-PA) is the only current effective treatment of acute ischemic stroke, but it is restricted to the first few hours after disease onset [48]. The utility of current platelet aggregation inhibitors and anticoagulants is counterbalanced by the risk of intracerebral bleeding complications, and the development of novel antiplatelet agents with a more favorable safety profile, better efficacy and rapid action in acute events remains a challenge. After the interruption of cerebral blood flow, tissue injury begins with an inflammatory reaction, which is a common response of the cerebral parenchyma to various forms of insult. Moreover, not only ischemia, but also reperfusion in itself causes tissue injury. Infiltrating leukocytes, especially neutrophils, play a pivotal role in propagating oxidative stress-triggered tissue damage after cerebral ischemia and reperfusion [50].

In a mouse model of acute ischemic stroke (tMCAO), it appears that platelets contribute to stroke progression by mechanisms that at least partially differ from those involved in thrombus formation [51,52]. Indeed, inhibiting early steps of platelet adhesion and activation (*i.e.*, VWF-GPIb, collagen-GPVI), but not aggregation (αIIbβ3 inhibitors), reduces infarct size. Platelets serve pro-inflammatory functions that are likely involved in infarct growth.

However, the mechanistic links between platelets and inflammation remain largely unknown. Our recent experimental data indicate that P2X1 receptors expressed on both platelets and neutrophils may represent such a link. It would therefore be interesting to determine whether defective thrombus formation observed in the microcirculation of P2X1−/− mice would protect these mice from thrombo-inflammatory ischemic brain infarction.
